# Towards Highly Efficient Nitrogen Dioxide Gas Sensors in Humid and Wet Environments Using Triggerable-Polymer Metasurfaces

**DOI:** 10.3390/polym15030545

**Published:** 2023-01-20

**Authors:** Octavian Danila, Barry M. Gross

**Affiliations:** 1 Physics Department, University Politehnica of Bucharest, 060042 Bucharest, Romania; 2 Optical Remote Sensing Laboratory, The City College of New York, New York, NY 10031, USA; 3 NOAA—Cooperative Science Center for Earth System Sciences and Remote Sensing Technologies, New York, NY 10031, USA

**Keywords:** metasurfaces, gas sensors, frequency-selective surface, optical sensing

## Abstract

We report simulations on a highly-sensitive class of metasurface-based nitrogen dioxide (NO${}_{2}$) gas sensors, operating in the telecom C band around the 1550 nm line and exhibiting strong variations in terms of the reflection coefficient after assimilation of NO${}_{2}$ molecules. The unit architecture employs a polymer-based (polyvinylidene fluoride—PVDF or polyimide—PI) motif of either half-rings, rods, or disks having selected sizes and orientations, deposited on a gold substrate. On top of this, we add a layer of hydrophyllic polymer (POEGMA) functionalized with a NO${}_{2}$-responsive monomer (PAPUEMA), which is able to adsorb water molecules only in the presence of NO${}_{2}$ molecules. In this process, the POEGMA raises its hidrophyllicity, while not triggering a phase change in the bulk material, which, in turn, modifies its electrical properties. Contrary to absorption-based gas detection and electrical signal-based sensors, which experience considerable limitations in humid or wet environments, our method stands out by simple exploitation of the basic material properties of the functionalized polymer. The results show that NO${}_{2}$-triggered water molecule adsorption from humid and wet environments can be used in conjunction with our metasurface architecture in order to provide a highly-sensitive response in the desired spectral window. Additionally, instead of measuring the absorption spectrum of the NO${}_{2}$ gas, in which humidity counts as a parasitic effect due to spectral overlap, this method allows tuning to a desired wavelength at which the water molecules are transparent, by scaling the geometry and thicknesses of the layers to respond to a desired wavelength. All these advantages make our proposed sensor architecture an extremely-viable candidate for both biological and atmospheric NO${}_{2}$ gas-sensing applications.

## 1. Introduction

Metasurfaces are artificial materials that have the ability to influence and control the electromagnetic field by means of almost any degree of freedom in their construction [1,2]. As a result, a significant number of effects have been observed, some of them recreating the ones in bulk materials and some completely new. The effects that can also be observed in conventional bulk materials are spectral filtering by absorption [3,4,5], and wavefront and polarization control [6,7,8,9,10]. New, ’exotic’ effects that were reported include electromagnetic cloaking [11,12,13], generalized reflection/refraction [14,15,16], perfect absorption via anisotropy [17], and enhanced emission by means of epsilon-near-zero materials [18]. Initially, metasurface architectures consisted of a unit cell with a certain pattern: metallic rods, rings, crosses, C-shapes, V-shapes, Y-shapes, or circular and elliptical disks, made of either gold, silver, copper, beryllium, and aluminum were deposited on either a silicon or a silicon oxide (SiO${}_{2}$) substrate and illuminated with a certain electromagnetic field in the spectral window of interest. The metallic nanostructures favored the creation of surface plasmons which oscillated at a resonance frequency, providing on-demand absorption and spectral filtering. However, for some optical and terahertz applications, such architectures exhibited significant losses in the metallic layers, which, together with heat dissipation and subsequent dilation, offered a relatively unstable resonant behavior. This issue was addressed by the creation of hybrid (metallic-semiconductor) [19] and all-dielectric metasurface architectures [20,21]. In the case of some all-dielectric metasurfaces, the materials and geometries chosen generate a dielectric permittivity that follows a hyperbolic dispersion rather than an elliptical one [22,23], which leads to new ways of manipulating the wavefronts of the reflected and transmitted waves. More recently, adaptive metasurfaces using externally-addressable materials have opened the way for active tuning and increased versatility towards the spectral domain required by the application [24,25,26]. Metasurfaces can also exhibit sensitivity to certain molecules deposited on them, which can induce a variation in their electric or magnetic properties, leading to a perturbed response with respect to an ideal case. This arrangement generates the possibility to realize both biological [27,28] and atmospheric gas sensors. Of the latter, the broadest attention has been dedicated to the development of CO${}_{2}$ gas sensors, which can be obtained by layering a thin, CO${}_{2}$ sensitive polymer on top of the metasurface structure [29] or by mounting the whole metasurface on a resistive heater and observing the absorbed thermal emission [30]. By comparison, much less attention has been dedicated to the high-sensitivity detection of nitrogen dioxide (NO${}_{2}$), which is a direct result of fuel combustion, and considered a pollutant gas in higher concentrations. In nature, NO${}_{2}$ composes the ozone layer in the Earth’s troposhpere and creates low-concentration nitrate aerosols. While not as efficient as CO${}_{2}$ or other nitrous oxides (such as N${}_{2}$O), NO${}_{2}$ can also have positive radiative forcing effects, making it a greenhouse gas [31]. Recently, a class of NO${}_{2}$-responsive polymers, such as N-(2-aminophenyl) methacrylamide hydrochloride (NAPMA) [32,33], as well as poly(oligo(ethylene glycol) methyl ether methacrylate (POEGMA), functionalized with endogenous NO-monomers, such as poly(2-(3-(2-aminophenyl) ureido)ethyl methacrylate) (PAPUEMA) [34], have been reported. Under the presence of NO${}_{2}$ molecules, the two polymers have been shown to exhibit strong hydrophylic behaviors which, in turn, in the presence of water molecules, induces high gradients in their electric properties such as dielectric permittivity and conductivity [35,36].

In this paper, we report simulations on a metasurface-based nitrogen dioxide gas detector based on a metasurface architecture layered with a PAPUEMA-functionalized POEGMA polymer, tailored to increase their sensitivity to NO${}_{2}$ molecules by means of the change in electric permittivity. We model the electric properties of the polymer layers when used in conjunction with the metasurface architecture and highlight the frequency response around the central wavelength $\lambda_{0}=1.55\;\mu$m, in the presence of NO${}_{2}$ and as a function of humidity in the proximity of the sensor. The selection of this spectral window was made for two reasons: firstly, the laser beam is not attenuated by the water droplets in high-humidity conditions, and, secondly, the telecom C band around 1550 nm has a multitude of off-the-shelf components which can be used to create low-cost gas sensors. Recent reports [37], in which InP nanowire arrays are used to monitor NO${}_{2}$ at room temperature by measuring an electrical signal have provided a significant level of insight in the realization of NO${}_{2}$ gas sensors in typical ambient environments. Our method differs from the former due to the fact that our sensor is designed for use in humid and wet environments, where the recording of small-level electrical signals can become challenging. The simulation results show that, when appropriately tuned, the architecture shows enhanced detection sensitivity for NO${}_{2}$ molecules in high-humidity conditions. This makes our proposed architecture suitable for a series of biological and atmospheric NO${}_{2}$ sensors which use humidity to their advantage, instead of considering it a parasitic effect.

## 2. Design Considerations

**Figure 1:**
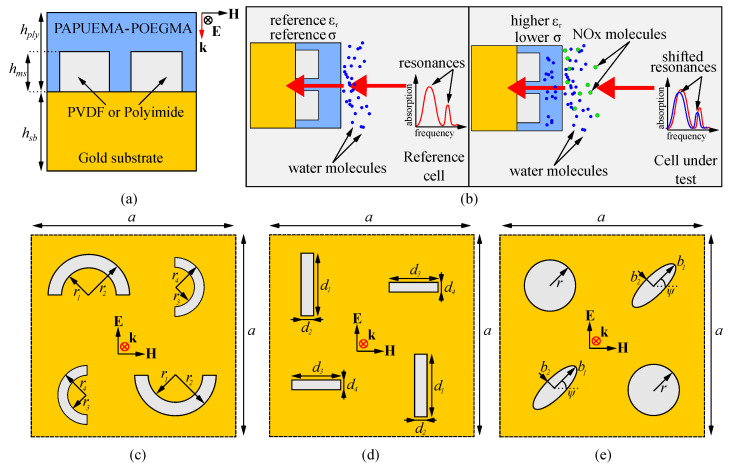
The structure and operating principles of the metasurface-based NO${}_{2}$ gas sensors: (**a**) the layers comprising the architecture with their respective thicknesses; (**b**) example of operating the reference and test metasurfaces: the reference cell (left side) is operated in the absence of NO${}_{2}$ molecules, and the PAPUEMA-POEGMA layer exhibits reference values of the relative permittivity $\epsilon_{r}$ and electric conductivity σ. The test cell (right side) is operated in the presence of NO${}_{2}$ molecules, which increase the hydrophyllic state of the PAPUEMA-POEGMA and considerably increase both $\epsilon_{r}$ and σ. The absorption readout of the cell under test is modified considerably with respect to the reference cell, usually in the form of prominent absorption peaks, if the reference is set to a fully-reflective behavior; (**c**) the incomplete ring metasurface layout; (**d**) the rod metasurface layout; and (**e**) the cylinder metasurface layout.

All our envisioned architectures follow the following layering scheme: a polymer-based metasurface having thickness $h_{ms}$ similar to the penetration depth of the incoming radiation is deposited on a gold substrate having thickness $h_{sb}$ much larger than the penetration depth of the incoming radiation. It should be noted that the penetration depth for mid-infrared radiation is $h_{pd}≃0.2\;\mu$m. On top of this, another NO${}_{2}$-sensitive polymer layer having $h_{ply}$ is deposited. For our study, we chose the POEGMA polymer functionalized with a NO${}_{2}$-reactive monomer known as PAPUEMA [34]. The thickness of the polymer represents a degree of freedom which is subject to optimization: as the thickness increases, so does the sensitivity to NO${}_{2}$ molecules, however, as the thickness reaches several times the penetration depth, the interaction with the metasurface pattern layered underneath it is no longer possible, and the whole architecture attains the behavior of the upper surface of the NO${}_{2}$-sensitive polymer layer [38]. The layering scheme is presented in Figure 1a. Assuming near-optimal polymer layer thickness, the operating principle of the NO${}_{2}$-based sensor is the following: in the reference configuration, the whole architecture is placed in a humid environment which lacks any NO${}_{2}$ molecules. This allows the upper layer to absorb trace amounts of water molecules, which contribute to the baseline levels of its dielectric permittivity and electric conductivity. The architecture is illuminated with mid-infrared radiation, which for our case was the doubled CO${}_{2}$ laser line operating at $\lambda_{0}=5.8\;\mu$m, and the absorption and reflection spectra are recorded. Due to the large thickness of the gold layer, the transmission through the sample is considered negligible. When performing measurements in an environment with NO${}_{2}$ molecules, their adsorption by the PAPUEMA monomer will trigger a change in the hydrophyllic nature of the POEGMA component layer, allowing it to absorb a considerably-larger amount of water molecules, and, therefore, change its electric parameters over a large scale of values. In the case of POEGMA, the reported change in the values of the relative permittivity was from 8.04 in a ’quasi-dry’ state to almost 63 in a ’wet’ state [36], accompanied by an increase in its electric conductivity by at least one order of magnitude. The absorption spectra obtained for the cell under test is always compared to the one of the reference cell. The operating principle is presented in Figure 1b. The metasurface architecture extends the range of basic resonator shapes previously reported in [39,40] to incomplete rings, rods, and cylinders fabricated out of polyvinylidene fluoride (PVDF) or polyimide (PI). The geometry comprises of a super-cell of unit geometries (either rings, rods, or cylinders) with different sizes spread symmetrically across the square surface of the super-cell having linear size *a*. The layouts are presented in Figure 1c for the incomplete rings, Figure 1d for the rods and Figure 1e for the cylinders. The geometric sizes are specific to each architecture so that they produce a significant response in the desired spectral window, and their coordinates (x,y) across the cell are set in such a way that the centers of the shapes are located in points (a/4,3a/4), (3a/4,3a/4), (3a/4,a/4), and (a/4,a/4), respectively, to preserve a degree of symmetry to the design.

In terms of material properties, we considered the architecture non-responsive to magnetic fields (i.e., $\mu_{r}=1$). Due to the fact that the architecture is subjected to high-frequency radiation, the DC values of the dielectric permittivity and electric conductivity have to be adjusted for the gold substrate. In the study, we have considered a Drude conduction and relaxation model, with DC conductivity $\sigma_{Au,DC}=4.517\times 10^{7}$ S/m. The Drude model then yields an effective conductivity for the gold substrate [41]:

(1) $$\sigma_{Au,eff}=\frac{\sigma_{0}}{1+j\omega \tau }$$

where $j=\sqrt{-1}$ is the imaginary unit, ω=2πf is the angular frequency associated to the radiation frequency *f*, and τ≃27 fs is the average relaxation time of the gold atoms. From this, assuming the effective relaxation-effect model, the relative permittivity can be calculated as [41]:

(2) $$\epsilon_{r,Au,eff}=\frac{1}{j\omega \epsilon_{0}}\left(\sigma^{\prime }+j\left(\omega \epsilon_{0}-\sigma "\right)\right)$$

where $\sigma^{\prime }=Re\left\{\sigma \right\}$ and $\sigma "=Im\left\{\sigma \right\}$ are the complex components of the electric conductivity, and $\epsilon_{0}$ is the vacuum permittivity. For dielectrics, the relaxation time is increased from femtoseconds to nanoseconds, and, therefore, the classical “skin-effect” model applies. In this assumption, the conductivity and permittivity values at THz frequencies can be approximated by the DC or low frequency (kHz to MHz) values. The DC/MHz values for the PVDF and PI polymer elements are recreated from previous works [26,42], and the baseline properties of the POEGMA layer are extracted from [36]. The electrical properties are centralized in Table 1.

To express the variation of POEGMA’s dielectric constant and electric conductivity as a function of the trigger rate resulting from the action of the NO${}_{2}$ molecules, we assume a linear dependence of the form:

(3) $$\begin{array}{cc} \epsilon_{r}=\epsilon_{r,dry}+\beta_{\epsilon }\gamma w & \sigma =\sigma_{dry}-\beta_{\sigma }\gamma w \end{array}$$

where $\gamma =N_{x}/N_{w}$ is the molar ratio between the NO${}_{2}$ and water molecules in the interaction volume close to the PAPUEMA-POEGMA layer, and *w* is the air humidity expressed in parts per million (ppm). In the ’wet’ state, where we assume w=1, the reported dielectric constant is 68 [36], the calculated permittivity wetting factor $\beta_{\epsilon }$ is calculated at 59.96 ppm${}^{-1}$. Upon wetting, the conductivity of the POEGMA layer decreases, which is consistent with the increase in permittivity. We also assume a linear dependence $\sigma =\sigma_{dry}-\beta_{\sigma }w$. In the ’wet’ state, the conductivity decreases by a factor of almost 20, which leads to a a conductivity wetting factor $\beta_{\sigma }=1.9\times 10^{-8}$ S·m${}^{-1}\cdot$ppm${}^{-1}$. To model the ’triggering’ effect of the wetting in the presence of NO${}_{2}$, we assume that the chance for a NO${}_{2}$-mediated water molecule absorption is proportional to the instant number of NO${}_{2}$ molecules around the POEGMA layer, modified by the triggering efficiency α of a NO${}_{2}$ molecule with the layer. This leads to an exponential rate of triggering, similar to any population rate equation:

(4) $$P\left(n\right)=C\exp\left(\alpha n\right)$$

where *n* is the instant number of NO${}_{2}$ molecules in close proximity of the POEGMA layer. Assuming that the probability of triggering an absorption is close to unity at some large number *N* of NO${}_{2}$ molecules, the normalizing constant *C* becomes:

(5) $$\begin{array}{c} C≃{\left(\int_{0}^{N}\exp\left(\alpha n\right)dn\right)}^{-1}=\frac{\alpha }{\exp\left(\alpha N\right)-1} \end{array}$$

Following the two relations above, for n≃N sufficiently large, the trigger rate then becomes $P\left(n\right)≃\alpha$. This is indeed the case for regular atmospheric concentrations of NO${}_{2}$ in urban areas, where the typical values of 50 ppb (parts per billion) is approximately $N_{x}=3\times 10^{16}$ NO${}_{2}$ molecules per mole of air. In our simulations, this value corresponds to 100% humidity. To model imperfect NO${}_{2}$ triggering, we chose conservative values of α at 25% as the NO${}_{2}$ trigger rate. In dry air, the water vapor concentration is approximately zero, whereas in humid air, the concentration can reach w=15,740 ppm [43], corresponding to $N_{w}=9.48\times 10^{21}$ water molecules per mole of air. Combining all the considerations performed under all the assumptions above, the net variation in the values of the electrical parameters in the presence of NO${}_{2}$ is:

(6) $$\begin{array}{cc} \epsilon_{r}=\epsilon_{r,dry}+\left(\beta_{\epsilon }\gamma w\right)\alpha ; & \sigma =\sigma_{dry}-\left(\beta_{\sigma }\gamma w\right)\alpha \end{array}$$

Within this framework, the variation of humidity from zero to one produces variations of the electric permittivity function at the second decimal, however, due to the subwavelength nature of the architecture, this variation is enough to produce significant variations in the response. This increased sensitivity is key in the design and potential applications of our proposed considerations. It is worth mentioning, however, that real implementations of such architectures suffer both from imperfections in the geometrical sizes of the elements, thickness of the layers, and induced trigger rates from the nitrogen dioxide molecules. Moreover, the duty-cycle of any potential sensors relying on this architecture will have to take into account imperfect drying cycles of the PAPUEMA-POEGMA polymer layer, which may lead to long-term deviations from the reference response. These real-life situations, however, can be accounted for with initial and periodic calibration of the sensor, in conjunction with the recording of the device’s behavior of the device across multiple wet-dry cycles. Variations in the geometric response affect the response by shifting the resonance peaks from that particular wavelength. Small variations (5–10%) from the reference configuration provide an almost linear shift in the resonance peaks, as indicated by previous studies in polymeric metasurfaces [26]. Depending on the sizes of the metasurface, the shift can be compensated by using any readily available tunable laser source operating in the telecom C-band. Most state-of-the-art nano-imprint and deposition techniques are able to operate within this tolerance. For larger variation, a calibration of the sensor using a wideband spectrometer setup is needed. To account for wetting–drying cycles, we use the results obtained in Ref. [36], in which the PAPUEMA-POEGMA retained water droplets equivalent to 5% humidity over multiple wet–dry cycles. The results obtained indicate that the variation in the resonant response at 5% humidity are negligible with respect to the reference configuration.

In terms of simulation conditions, we used a finite element method (FEM)-based commercial solution, namely COMSOL Multiphysics, RF Module. The solution allows for accurate simulation with iterative error estimation. Meshing of the complete simulation environment was set to a size of $\lambda_{0}/20$, where $\lambda_{0}=1.55\;\mu$m is the central working wavelength, with an increase in the spatial resolution of narrow regions to nanometer size. The accuracy of the iterative solver was increased in such a way that any solution was considered accurate if the estimated error was below $10^{-6}$. To simulate the periodicity of the unit cell, all side walls of the simulation cell were set with Floquet periodicity, and a Floquet wave-vector that was directly taken from the simulation port. Modeling the skin depth of the PVDF and PI elements was performed by attributing a transition boundary condition on the interface between the elements and the gold substrate. Additionally, to save computing time, the back plate of the gold substrate was set to an impedance boundary condition, since no transmission occurs through the architecture.

## 3. Results and Discussion

Regardless of the configuration used, the first step of our investigation was tuning the geometric sizes and layer thicknesses in such a way as to achieve a significant response close to $\lambda_{0}$ as possible, when the metasurface is in its reference configuration (i.e., dry environment). The spectral behavior was quantified by means of measuring the logarithmic $S_{11}$ parameter (negative return loss factor), defined as:

(7) $$S_{11}=10\log\left(\frac{P_{refl}}{P_{in}}\right)$$

where $P_{in}$ and $P_{refl}$ are the input and reflected power levels, respectively. We remind that, typically, the $S_{11}$ parameter is negatively-valued. A 0 dB value of the $S_{11}$ parameter is equivalent to a fully-reflective interface (no return loss), while a negative value represents partial absorption of the input field (positive return loss value). The representation of $S_{11}$ allows more sensitivity in the determination of absorption peaks when compared to standard optical visualizations. Finally, for each situation, we envision an application scenario, where the metasurface is used as a NO${}_{2}$ gas detector. In these scenarios, the change in response can be quantified via two methods: the first method, known as the wavelength interrogation method, assesses the shift of a certain resonance peak as a function of the increase in humidity levels. The second, known as the signal level interrogation method, assesses the change in the level of the $S_{11}$ parameter at a single frequency as a function of increasing humidity levels. Given a reference state *p* and a measured state *q*, each method comes with its own average figure of merit, defined as the rate of change of the interrogated physical quantity (i.e., wavelength or reflection coefficient) as a function of the applied quantity (i.e., humidity level or polarization state) between the two states. Representing as a function of humidity factor *w*, the figures of merit characterizing the deviation from reference are defined as:

(8) $$\begin{array}{cc} FOM_{\lambda }^{\left(pq\right)}\left(w\right)=\frac{\lambda_{q}-\lambda_{p}}{w_{q}-w_{p}}; & FOM_{S}^{\left(pq\right)}\left(w\right)=\frac{S_{q}-S_{p}}{w_{q}-w_{p}}{|}_{\lambda_{0}} \end{array}$$

with the same formulas being used for the interrogation methods as a function of the change in state of polarization for the input field.

### 3.1. Half-Ring Architecture

**Figure 2:**
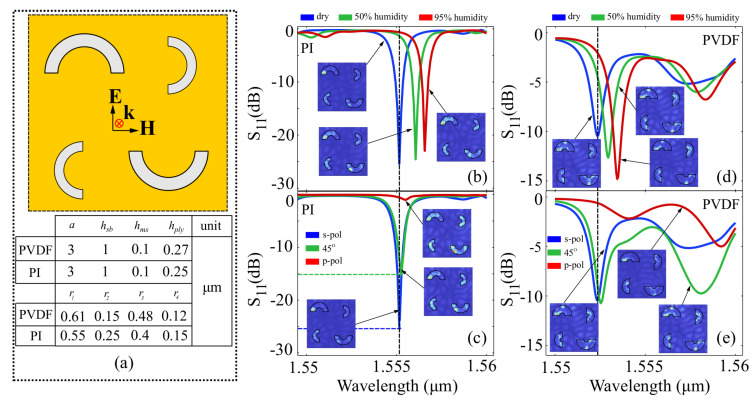
Results obtained for the half-ring structure: (**a**) layout of the ring elements and element sizes chosen for the PVDF and PI in order to have a resonant response around the working wavelength; (**b**) spectral behavior of the PI half-ring element structure at different humidity levels and (**c**) at different input polarization states; and (**d**) spectral behavior of the PVDF half-ring element structure at different humidity levels and (**e**) at different input polarization states. For subfigures (**b**–**e**), the insets show the local field enhancement in the interface plane taken at maximum resonance.

The results for the half-ring structure are presented in Figure 2, in which a strongly resonant response in the desired window is evident, followed by a significant modification in the metasurface behavior as a function of external humidity factor and input field polarization.

Based on the results obtained above, in the reference configuration shown in Figure 2b, the PI-element metasurface exhibits a strong resonance at 1555 nm, with a 26 dB return loss at the input port, which is well below the 1% reflection threshold. In the presence of NO${}_{2}$ molecules and 50% external humidity, the resonance peak is shifted towards 1557 nm, and the return loss moves to 23 dB, whereas in the case of 95% external humidity, the resonance peak is shifted at 1558 nm, with a 22 dB return loss. The bandwidth of the resonances is roughly 0.8 nm, which implies that in applications, the spectrum can be readily sampled with off-the-shelf narrowband filters, some of which are exhibiting less than 50 picometer resolution. Assuming level detection is performed via wavelength interrogation, the figures of merit for the wavelength interrogation method are $FOM_{\lambda }^{\left(21\right)}\left(w\right)=1.016$ nm·ppm${}^{-1}$ for the dry-to-50% humidity increase scenario and $FOM_{\lambda }^{\left(31\right)}\left(w\right)=1.021$ nm·ppm${}^{-1}$ for the dry-to-95% humidity increase scenario. For the signal level interrogation method, the figures of are $FOM_{S}^{\left(21\right)}\left(w\right)=3.2\times 10^{-3}$ dB·ppm${}^{-1}$ for the zero-to-50% humidity increase scenario, and $FOM_{S}^{\left(31\right)}\left(w\right)=3.6\times 10^{-3}$ dB·ppm${}^{-1}$. When changing the input field polarization, the PI-element metasurface exhibits high sensitivity and low morphing behavior, preserving its absorption peak at 1555.2 nm. Assuming the same behavior in terms of humidity increase, the preservation of the wavelength corresponding to the resonance implies that the wavelength interrogation method can be reliably used with unpolarized radiation. In terms of signal level interrogation, the polarization figures of merit are $FOM_{S}^{\left(21\right)}\left(SOP\right)=15.27$ dB·rad${}^{-1}$ for a $45^{\circ }$ polarization state rotation, and $FOM_{S}^{\left(31\right)}\left(SOP\right)=15.91$ dB·rad${}^{-1}$ for the $90^{\circ }$ rotation. For the PVDF element, the chosen geometry provides a resonance response at 1552.5 nm in the reference configuration. As depicted in Figure 2d, the increase in humidity shifts the resonance peak to 1553.5 nm in the case of 50% humidity and to 1554.3 nm for 100% humidity. The corresponding figures of merit are $FOM_{\lambda }^{\left(21\right)}\left(w\right)=1.27\times 10^{-4}$ nm·ppm${}^{-1}$ for the zero-to-50% humidity scenario, and $FOM_{\lambda }^{\left(31\right)}\left(w\right)=1.2\times 10^{-4}$ nm·ppm${}^{-1}$ for the zero-to-100% humidity scenario. Regarding signal-level interrogation taken at reference configuration resonance frequency, the signal increases from −11 dB to −4 dB and −2.5 dB for the 50% and 100% humidity scenarios, respectively. The corresponding figures of merit are $FOM_{S}^{\left(21\right)}\left(w\right)=8.9\times 10^{-4}$ dB·ppm${}^{-1}$ for the dry-to 50% humidity shift, and $FOM_{S}^{\left(31\right)}\left(w\right)=6.35\times 10^{-4}$ dB·ppm${}^{-1}$ for the dry-to-95% humidity. In terms of polarization behavior, shown in Figure 2e, the PVDF-element metasurface is sensitive to input polarization in the reference configuration. The resonance peak at 1552.5 nm does not shift, however it is reduced drastically when the input polarization state is close to p-polarization. The structure also exhibits a second resonance for an input polarization of $45^{\circ }$, which reaches −10 dB at around 1557.5 nm. Since the morphing behavior of the spectrum is quite strong, a wavelength-based figure of merit does not provide any relevant information, since resonance peaks are not preserved for all polarization states. For a single wavelength, however, the signal level interrogation method can still be applied. Taking the reference resonance peak wavelengths, the figures of merit are $FOM_{S}^{\left(21\right)}\left(SOP\right)=-0.127$ dB·rad${}^{-1}$ for a $45^{\circ }$ rotation, and $FOM_{S}^{\left(31\right)}\left(SOP\right)=6.04$ dB·rad${}^{-1}$. As a common property of all half-ring structures under study, it can be seen that regardless of the variational parameter (humidity or input field polarization) the local field enhancement maps in the insets show a localization of the oscillating plasmon in the ring structure, which confirm the strong resonances observed.

### 3.2. Rod Architecture

**Figure 3:**
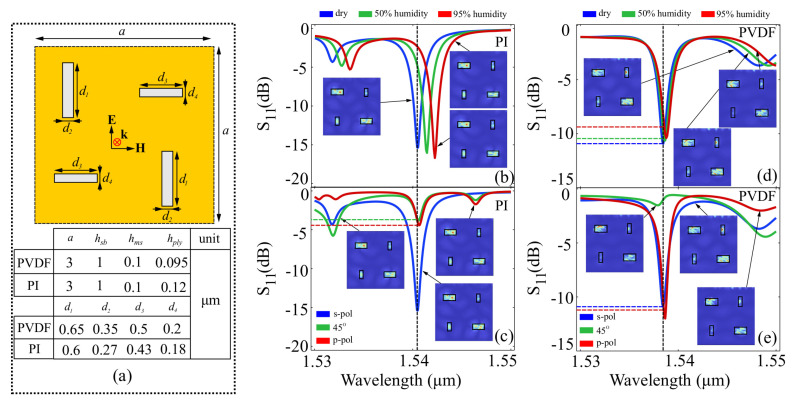
Results obtained for the rod-based structure. (**a**) Layout of the ring elements and element sizes chosen for the PVDF and PI in order to have a resonant response around the working wavelength; (**b**) spectral behavior of the PI rod-element structure at different humidity levels and (**c**) at different input polarization states; and (**d**) spectral behavior of the PVDF rod-element structure at different humidity levels and (**e**) at different input polarization states. For subfigures (**b**–**e**), the insets show the local field enhancement in the interface plane taken at the resonance peak.

In the case of the rod architecture, the PVDF and PI elements were arranged in an equivalent manner to the half-rings. The structure and element sizes are presented in Figure 3a.

Simulations performed on the PI element structure indicate that for an appropriate element size and analyte height configuration, a strong resonance, with a return loss of 15.3 dB is observed at 1540.5 nm, in the reference configuration. The resonance is reasonably narrow, with a FWHM bandwidth of approximately 1.5 nm. When increasing external humidity to 50%, the resonance peak shifts towards 1542 nm, while maintaining its FWHM, and attaining a return loss of 16 dB. For 95% humidity, the peak is shifted towards 1543 nm, with an associated return loss of 16.5 dB and negligible FWHM modification. The response is presented in Figure 3b. When applying the two interrogation methods, the figure merits are calculated as $FOM_{\lambda }^{21}\left(w\right)=1.9\times 10^{-4}$ nm·ppm${}^{-1}$ and $FOM_{\lambda }^{31}\left(w\right)=1.67\times 10^{-4}$ nm·ppm${}^{-1}$ for the resonance peak interrogation method, and $FOM_{S}^{\left(21\right)}\left(w\right)=0.88\times 10^{-4}$ dB·ppm${}^{-1}$ and $FOM_{S}^{\left(31\right)}\left(w\right)=0.88\times 10^{-4}$ dB·ppm${}^{-1}$. For polarization sensitivity measurements of the reference configuration, presented in Figure 3c, the resonances have negligible shifts, however, the signal suffers strong return loss attenuation, from −15.3 dB to −4.6 dB in the case of a $45^{\circ }$ rotation, and −4.1 dB in the case of a $90^{\circ }$ rotation. The calculated figures of merit are: $FOM_{\lambda }^{\left(21\right)}\left(SOP\right)≃FOM_{\lambda }^{\left(31\right)}\left(SOP\right)<10^{-6}$ nm·rad${}^{-1}$. Signal interrogation figures of merit, however, are $FOM_{S}^{\left(21\right)}\left(SOP\right)=13.62$ dB·rad${}^{-1}$ for a $45^{\circ }$ rotation, and $FOM_{S}^{\left(31\right)}\left(SOP\right)=14.26$ dB·rad${}^{-1}$ for a $90^{\circ }$ rotation. For the PVDF-element architecture, the effect of humidity in shifting the resonance peaks is relatively negligible, as presented in Figure 3d. The resonance peak shifts from 1539.2 nm to 1539.25 nm for an increase in humidity from zero to 50%, and from 1539.2 nm to 1539.27 nm for an increase in humidity from zero to 95%. The associated figures of merit for the resonance wavelength interrogation method are $FOM_{\lambda }^{\left(21\right)}\left(w\right)≃FOM_{\lambda }^{\left(31\right)}\left(w\right)<10^{-6}$dB·ppm${}^{-1}$ for both humidity shift scenarios. In terms of signal interrogation, the return loss factor varies from −10.8 dB to −10.3 dB for the dry-to-50% humidity shift and to −9.3 dB for the dry-to-95% humidity shift, respectively. The associated figures of merit are $FOM^{\left(21\right)}=6.35\times 10^{-5}$ dB·ppm${}^{-1}$ for the dry-to-50% humidity shift, and $FOM^{\left(31\right)}=1.002\times 10^{-4}$ dB·ppm${}^{-1}$ for the dry-to-95% humidity shift. The polarization-dependent response of the PVDF presented in Figure 3e exhibits significant changes. Specifically, for a horizontal and vertical input *SOP*, the behavior of the metasurface remains unchanged, with a negligible shift in the resonance peak, and a slight signal level modification. However, for a $45^{\circ }$ SOP, the response has a relatively-small resonance peak, which also exhibits Fano-like asymmetry. For the wavelength interrogation method, the figures of merit are virtually negligible, as the resonance peak moves only slightly. For the signal level interrogation method, due to the fact that the response of the metasurface is not changing monotonously across the polarization spectrum, the figures of merit do not offer relevant information. The insets of Figure 3b–e also show the local field enhancement in the interface plane taken at the resonance peak. It can be seen that for the majority of cases, the electric field enhancement is localized in the PI and PVDF elements, which is in total agreement with theoretical models regarding dielectric resonators.

### 3.3. Disk Architecture

**Figure 4:**
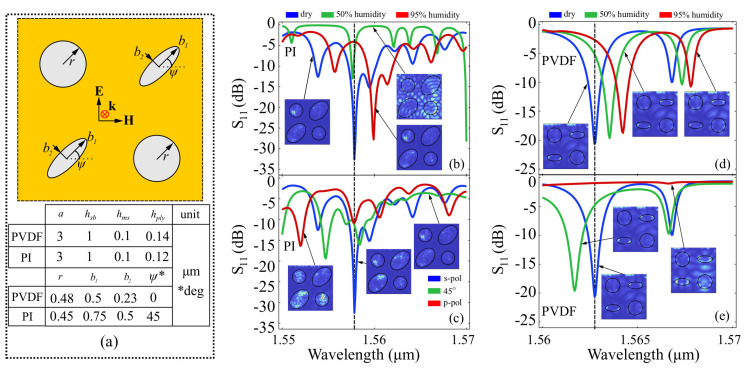
Results obtained for the disk-element architecture. (**a**) Layout of the ring elements and element sizes chosen for the PVDF and PI in order to have a resonant response around the working wavelength; (**b**) spectral behavior of the PI disk-element structure at different humidity levels and (**c**) at different input polarization states; and (**d**) spectral behavior of the PVDF disk-element structure at different humidity levels and (e) at different input polarization states. For subfigures (**b**–**e**), the insets show the local field enhancement in the interface plane taken at the resonance peak.

Just as before, we have adjusted the dimensions of the disk elements so that resonances are produced in the desired spectral window. The structure and element sizes are presented in Figure 4a.

For the PI disk-element architecture, we observe a strong resonance peak at 1558.6 nm, with a return loss of 32.5 dB. As it can be inferred from Figure 4b. The spectral response retains its shape when varying the humidity factor, with an associated shift in the resonance peaks which does not exhibit monotony. When increasing humidity to 50%, the resonance peak does not shift, and only has its return loss attenuated to 12.5 dB. When increasing humidity to 95%, the resonance peak shifts to 1560 nm, and the shift is associated with a return loss factor of 28.5 dB. The polarization-dependent response, shown in Figure 4c, shows that the reference configuration suffers considerable modifications when the SOP is switched to $45^{\circ }$ and $90^{\circ }$, respectively. For both SOP rotations, the spectrum becomes flatter, with resonances up to −15 dB, as well as shifted, with the highest resonances being observed at 1557 nm and 1552 nm, respectively. Due to the fact that the modification of the spectral response is not monotonous, calculating the figures of merit does not offer relevant information on the behavior of the metasurface. The humidity-dependent behavior of the PVDF disk-element architecture, presented in Figure 4d, exhibits a resonance peak at 1562.5 nm, and a highly-monotonous morphing behavior as a function of humidity. The main resonance peaks reaching −20 dB are shifted to 1563.9 nm, at 50% humidity, and to 1564.4 nm at 95% humidity. The response also exhibits another resonance peak reaching −10 dB at 1564 nm, which also has high morphing monotony as the humidity factor is increased. The figures of merit for the wavelength interrogation method are: $FOM_{\lambda }^{21}\left(w\right)=1.28\times 10^{-4}$ nm·ppm${}^{-1}$, and $FOM_{\lambda }^{31}\left(w\right)=1.06\times 10^{-4}$ nm·ppm${}^{-1}$. Assuming linearity in the shift, an average figure of merit associated to the resonance wavelength is $FOM_{\lambda }\left(w\right)=1.17\times 10^{-4}$ nm·ppm${}^{-1}$. The same technique can be applied to determine the figures of merit corresponding to the wavelength shift of the smaller resonance peak obtained in at 1565.2 nm for the reference configuration, and at 1565.4 nm and 1565.7 nm for the 50% and 95% humidity configurations, respectively. For the signal interrogation method taken at reference resonance wavelength, the signal varies significantly, from −20 dB in the reference configuration, to −6 dB and −3 dB in the 50% and 95% humidity configurations, respectively. The associated figures of merit are $FOM_{S}^{21}\left(w\right)=1.77\times 10^{-3}$ dB·ppm${}^{-1}$ and $FOM_{S}^{31}\left(w\right)=1.13\times 10^{-3}$ dB·ppm${}^{-1}$, with an average figure of merit $FOM_{S}\left(w\right)=1.45$ dB·ppm${}^{-1}$. When looking at the polarization picture, we observe a strong blueshift in the resonance when the reference metasurface is illuminated with a $45^{\circ }$ polarization, and virtually no resonance behavior when a vertical input polarization is used. The same behavior is observed for the smaller resonances at 1565.5 nm, where the resonance is either shifted or disappears completely, depending on the input polarization. Due to the fact that the wavelength shift is not monotonous in terms of polarization, a linear figure of merit cannot be defined. In terms of signal interrogation taken at the main resonance wavelength of the reference configuration, the resonance decreases from −20 dB to −5 dB in the case of the $45^{\circ }$ input polarization configuration, and to 0 dB in the case of the vertically polarized input field configuration. The associated figures of merit are: $FOM_{S}^{21}\left(SOP\right)=19.1$ dB·rad${}^{-1}$, and $FOM_{31}=12.73$ dB· rad${}^{-1}$, with an average figure of merit $FOM_{S}\left(SOP\right)=15.9$ dB· rad${}^{-1}$. The local field amplification taken at resonance frequencies and presented in the insets of Figure 4b–e show that for the majority of the cases, the local field is concentrated in the PI/PVDF elements, which supports theoretical assumptions regarding the appearance of resonances in such structures.

As a final note, in terms of angular and polarization stability, all envisioned architectures have been designed to operate under normal incidence of the input field, under linear polarization. A small variation in the incidence angle (less than 5${}^{\circ }$) produces negligible modification in the result. However, a small modification of the input polarization breaks the in-plane asymmetry, resulting in a high polarization sensitivity. This limitation can be overcome by providing a high-stability polarization laser via high-quality polarizers.

## 4. Conclusions and Outlook

In this paper, we have created and conducted simulations on a NO${}_{2}$ molecule-triggered responsive metasurface architecture in the near-infrared spectral domain around 1550 nm, which have the ability to use external humidity to its advantage. The architecture combines a metasurface layer made from a gold substrate with either a polyvinylidene fluoride (PVDF) or polyimide (PI) subwavelength element configuration, in the shape of either half-rings, rods or disks, with a NO${}_{2}$-responsive functionalized PAPUEMA-POEGMA layer. This layer has been proven to exhibit significant changes in its dielectric constant in the presence of NO${}_{2}$ molecules and humidity, by changing its hydrophyllic state. Owing to its functionalization with the PAPUEMA monomer, the POEGMA polymer is able to change its hydrophillic state only in the presence of NO${}_{2}$ molecules. The results obtained show that even in the conservative assumption of an imperfect trigger rate and small variation (at the second decimal) of the dielectric constant due to humidity increase, the subwavelength nature of the architecture is tuned to be extremely sensitive both to humidity increase and to polarization. To offer some rough quantitative characterization of the response modifications we introduced the wavelength and signal interrogation methods certain figures of merit, that, in the monotonous response variation assumption, offer information on the resonance shift and signal variation taken at the reference resonance. Real devices have to take into account, however any deviation from this ideal configuration, in terms of metasurface element and layer thickness size, relative displacement, as well as the imperfect restoration of the dielectric constant across multiple wetting and drying cycles. This impediment can be resolved, however, by conducting an initial and periodic calibration of the sensor thus designed. Overall, we believe the studies conducted here to be a promising stepping stone in the development of highly sensitive, low-cost NO${}_{2}$ sensors in the near-infrared telecom C band, around the 1550 nm wavelength.

## Figures and Tables

**Table 1:** Electrical properties of the polymer elements in the metasurface architecture.

$\epsilon_{r,\mathrm{PVDF}}$	$\sigma_{\mathrm{PVDF}}$ (S/m)	$\epsilon_{r,\mathrm{PI}}$	$\sigma_{\mathrm{PI}}$ (S/m)	$\epsilon_{r,\mathrm{POEGMA}}$	$\sigma_{\mathrm{POEGMA}}$ (S/m)
9.55−j×0.05	$1.01\times 10^{-3}$	3.13−j×0.02	$\left(2-j\times 0.01\right)\times 10^{-9}$	8.04−j×0.02	$1.5\times 10^{-10}$

## Data Availability

Not applicable.
